# Reperfusion Activates AP-1 and Heat Shock Response in Donor Kidney Parenchyma after Warm Ischemia

**DOI:** 10.1155/2018/5717913

**Published:** 2018-08-16

**Authors:** Alexandr Reznik, Olga Plotnikova, Andrey Skvortsov, Mikhail Skoblov, Oleg Reznik, Ancha Baranova

**Affiliations:** ^1^Department of Genetics, Atlas Biomed Group, Moscow, Russia; ^2^Moscow Institute of Physics and Technology, Moscow, Russia; ^3^Organ Transplant Department, First Pavlov State Medical University, Saint-Petersburg, Russia; ^4^Research Centre for Medical Genetics, Moscow, Russia; ^5^School of Biomedicine, Far Eastern Federal University, Vladivostok, Russia; ^6^Organ Procurement Center, St Petersburg Research Institute for Emergency Named after I.I.Dzanelidze, Saint-Petersburg, Russia; ^7^School of Systems Biology, George Mason University, Fairfax, VA, USA

## Abstract

Utilization of kidneys from extended criteria donors leads to an increase in average warm ischemia time (WIT), which is associated with larger degrees of ischemia-reperfusion injury (IRI). Kidney resuscitation by extracorporeal perfusion in situ allows up to 60 minutes of asystole after the circulatory death. Molecular studies of kidney grafts from human donors with critically expanded WIT are warranted. Transcriptomes of two human kidneys from two different donors were profiled after 35-45 minutes of WIT and after 120 minutes of normothermic perfusion and compared. Baseline gene expression patterns in ischemic grafts display substantial intrinsic differences. IRI does not lead to substantial change in overall transcription landscape but activates a highly connected protein network with hubs centered on Jun/Fos/ATF transcription factors and HSP1A/HSPA5 heat shock proteins. This response is regulated by positive feedback. IRI networks are enriched in soluble proteins and biofluids assayable substances, thus, indicating feasibility of the longitudinal, minimally invasive assessment* in vivo*. Mapping of IRI related molecules in ischemic and reperfused kidneys provides a rationale for possible organ conditioning during machine assisted ex vivo normothermic perfusion. A study of natural diversity of the transcriptional landscapes in presumably normal, transplantation-suitable human organs is warranted.

## 1. Introduction

Across a variety of transplanted organs, short-term patient and graft outcomes continue to improve [1], with 1-year survival rates for kidney recipients being well over 90% [2, 3]. However, improving longer-term outcomes remains a challenge [3].

In kidney transplantation, ischemia-reperfusion injury (IRI) is unavoidable. IRI contributes to both immunologically mediated chronic rejection [4] and so-called chronic allograft dysfunction (CAD) [5]. IRI, which is proportional to donor warm ischemia time (WIT), is one of the main factors influencing kidney graft survival [6]. Severity of renal IR is strongly associated with the circumstances of kidney donation [7]. Recent dramatic increase in the utilization of kidneys from donors after circulatory death and extended criteria donors lead to an increase in average WIT [8, 9]. It is widely accepted that the prevention or the reduction of IRI is imperative to improve graft survival and decrease posttransplant morbidity.

In transition between the donor and the recipient, renal allograft is typically preserved by static cold storage. Recently, there has been considerably increased interest in machine perfusion for preservation of kidneys, with meta-analysis showing that machine perfusion improves outcomes through the better preservation of tubular, glomerular, and endothelial function and integrity [10]. Recently, kidney resuscitation by extracorporeal perfusion in situ was added to the list of options, with up to 60 minutes of asystole allowed after the circulatory death [11].

High-throughput profiling technologies have enabled systemic investigation of the pathophysiological processes on the “omics” landscapes and subsequent molecular dissection of observed functional changes [12]. In previous studies, a number of mRNAs, miRNA, and proteins playing role in tubular or vascular damage to the donor organ were associated with the incidence and severity of IRI [13, 14]. However, no molecular studies so far were performed in kidney grafts from human donors with critically expanded WIT.

In this study, we analyzed the transcriptomes of two kidney grafts from two different donors. Grafts were biopsied after 40 and 49 minutes of WIT, respectively, and then after 120 minutes of normothermic perfusion. We showed that reperfusion does not lead to substantial change in overall landscape of kidney transcription but rather activates a specific program resulting in overexpression of highly connected protein network with hubs centered on Jun/Fos/ATF transcription factors and HSP1A/ HSPA5 heat shock proteins.

## 2. Materials and Methods

This study design, protocols of perfusion and samples preparation, organ procurement, and transplant procedures were approved by the Scientific Board and Ethics Committee of the Saint-Petersburg State Research Institute for Emergency (Decision 7/0615/09) and authorized for clinical application by the Federal Advisory Service of the Ministry of Healthcare of the Russian Federation (Resolution N2010/299). Both donors had unexpected irreversible asystole and circulatory death in course of their stay in the hospital. After unsuccessful attempts of advanced cardiopulmonary resuscitation, the entry to donor program was activated by “in-house” hospital transplant coordinator as described previously [11]. After the permission was obtained from hospital administration, donors were transferred to an operating room for femoral vessels catheterization and perfusion procedure setup. Simultaneously kidney parenchyma biopsies were taken using 20G needles (SuperCore II, Angiotech, USA) under ultrasound control. According to current legislation in Russia, informed consent from the relatives and next-of-kin for femoral access and for nonlaparotomic biospecimen collection is not required.

For both donors, in situ extracorporeal perfusion of an isolated abdominal region with membrane oxygenation and leukocyte depletion was performed. To prime the circuit, we used up to 2L of Custodiol™ (HTK, histidine-tryptophan-ketoglutarate solution, Dr. F. Kohler Chemie GmbH, Bensheim, Germany). Controlled organ reperfusion procedure consists of the following obligatory subprocedures:

1. Abdominal in situ thrombolysis and heparinization through perfusion circuit
2. Elimination of leuko- and thrombocyte clots from the vascular bed of abdominal organs using the hemodilution and leukofilter incorporated into perfusion circuit
3. Subnormothermic extracorporeal membrane oxygenation of the perfusate.

Organs were perfused with the modified donor blood augmented with the following: 1.5 million units of Streptokinase (Belmedpreparaty AO, Minsk, Belarus) and 25,000 IU of Heparin (Gedeon Richter, Hamburg, Germany). During the first 30 minutes of perfusion, the perfusate flow was gradually increased from 500 ml/min to 3500 ml/min. The oxygen supply was maintained at 150–350 ml/min which corresponds to an average pO2 of 300.1±9.38 mm Hg. All procedures were performed under mild normothermic or subnormothermic conditions (27–32°C). Blood samples were collected and assayed for leukocyte counts, pH levels, oxygen, and CO_2_. A count of 1×10^9^ or lower was empirically considered as a satisfactory perfusion outcome. On average, elimination of leukocytes from the abdominal perfusion circuit required no more than 120 minutes, the time that is sufficient to complete legal paperwork and obtain the next-of-kin consent.

Although the perfusion procedures were initiated prior to the arrival of the forensic pathologist, the organ procurement procedures started only after completion of legal documentation, including the consent of next-of-kin. Laparotomy and kidney mobilization were performed and organ recovery commenced while the donor was still on the continuous extracorporeal perfusion. The perfusion procedure was terminated just before the surgical kidney explantation, performed immediately after second needle biopsy. Each kidney graft was placed in a separate plastic bag for subsequent static cold preservation in HTK solution.

Four kidney grafts were subsequently transplanted into 4 patients according to approved protocol in Saint-Petersburg [11]. Prior to transplantation, all recipients signed informed consent to ensure their awareness of the study procedures performed with donor kidneys. In three of these patients, an immediate graft function was observed, while one had delayed graft function, which was restored after 6 sessions of hemodialysis. All four patients were routinely discharged after 21-day hospital stay. 3-year patients and grafts survival rates were at 100%. To date, one patient died of a stroke in the fifth year after transplant with functioning graft; one returned to kidney replacement therapy due to noncompliance in the fourth year after transplant. Two remaining patients are alive with functioning grafts and the most recent serum creatinine monitoring results at 98.7 and 101.0 mmol/L, respectively (test date: June 1^st^, 2018).

### 2.1. Kidney Donor Procedures

From 2011, Saint-Petersburg's Organ Procurement Center prospectively collects samples from brain dead donors and deceased donors enrolled throughout donation program. In this study, four kidney parenchyma specimens were collected from two different donors; one kidney in each pair was biopsied after 40 and 49 minutes of WIT, respectively, and after 120 minutes of normothermic perfusion. Biopsies were performed using 20G needles (SuperCore II, Angiotech, USA). Each parenchyma specimen was divided into 7 equal pieces, snap frozen into liquid nitrogen, and placed at -70°C for storage.

This study design, protocols of perfusion, organ procurement, and transplant procedures were approved by the Scientific Board and Ethics Committee of the Saint-Petersburg State Research Institute for Emergency (Decision 7/0615/09) and authorized for clinical application by the Federal Advisory Service of the Ministry of Healthcare of the Russian Federation (Resolution N2010/299). Both donors had unexpected irreversible asystole and circulatory death in course of their stay in the hospital. After unsuccessful attempts of advanced cardiopulmonary resuscitation, the entry to donor program was activated by “in-house” hospital transplant coordinator as described previously [11]. For both donors, in situ extracorporeal perfusion of an isolated abdominal region with membrane oxygenation and leukocyte depletion was performed. To prime the circuit, we used up to 2L of Custodiol (HTK, histidine-tryptophan-ketoglutarate solution, Dr. F. Kohler Chemie GmbH, Bensheim, Germany). Controlled organ reperfusion procedure consists of the following obligatory subprocedures:

(4) Abdominal in situ thrombolysis and heparinization through perfusion circuit
(5) Elimination of leuko- and thrombocyte clots from the vascular bed of abdominal organs using the hemodilution and leukofilter incorporated into perfusion circuit
(6) Subnormothermic extracorporeal membrane oxygenation of the perfusate.

Organs were perfused with the modified donor blood augmented with following: 1.5 million units of Streptokinase (Belmedpreparaty AO, Minsk, Belarus) and 25,000 IU of Heparin (Gedeon Richter, Hamburg, Germany). During the first 30 minutes of perfusion, the perfusate flow was gradually increased from 500 ml/min to 3500 ml/min. The oxygen supply was maintained at 150–350 ml/min which corresponds to an average pO2 of 300.1±9.38 mm Hg. All procedures were performed under mild normothermic or subnormothermic conditions (27–32°C). Blood samples were collected and assayed for leukocyte counts, pH levels, oxygen, and CO_2_. The decrease of leukocyte count in the perfusion circuit was used as an indirect indication to start the surgical recovery procedure. A count of 1×10^9^ or lower was empirically considered as a satisfactory perfusion outcome. On average, elimination of leukocytes from the abdominal perfusion circuit required no more than 120 minutes, the time that is sufficient to complete legal paperwork and obtain the next-of-kin consent.

Although the perfusion procedures were initiated prior to the arrival of the forensic pathologist, the organ procurement procedures started only after completion of legal documentation, including the consent of next-of-kin. Laparotomy and kidney mobilization were performed and organ recovery commenced while the donor was still on the continuous extracorporeal perfusion. The perfusion procedure was terminated just before the surgical kidney explantation. Each kidney graft was placed in a separate plastic bag for subsequent static cold preservation in histidine-tryptophan-ketoglutarate (HTK) solution (Essential Pharmaceuticals, LLC, Durham, USA).

### 2.2. Kidney Biopsy Procedures

From 2011, Saint-Petersburg's Organ Procurement Center prospectively collects samples from brain dead donors and deceased donors enrolled throughout donation program. In this study, four kidney parenchyma specimens were collected: two after 35-45 minutes of WIT and two after 120 minutes of normothermic perfusion. Each parenchyma specimen was divided into 7 equal pieces, snap frozen, and placed at -70°C for storage.

### 2.3. RNA Extraction and Library Construction

Total RNA was extracted from tissue specimens using Trizol reagent (Fisher Scientific, Hampton, USA) according to manufacture instruction. RNA quality was confirmed with BioAnalyser and RNA 6000 Nano Kit (Agilent, Santa Clara, USA). PolyA fraction of RNA was purified with Dynabeads® mRNA Purification Kit (Fisher Scientific, Hampton, USA). Illumina library was made from polyA RNA with NEBNext® mRNA Library Prep Reagent Set (NEB, Ipswich, USA) according to manual. Sequencing was performed on HiSeq1500 with average 50 bp read length for 10 million reads generated for each sample.

### 2.4. Transcriptome Bioinformatic Analysis

Initial quality control of sequencing outputs was performed using FastQC. The raw reads were mapped to the hg19 using the CLC Genomics Workbench 6.0.64 with a mismatch cost of 2 and controlled through generating Mapping Reports for each sample. For all the genes both Reads Per Kilobase of transcript per Million (RPKM) and total reads count were calculated. For each of the four libraries, RNAseq procedures generated about 8 mln reads, approximately 85% of which were effectively mapped to hg19. For each sample, a total of 32 860 genes were annotated.

The correlation analysis of gene expression values in two kidneys was performed by Pearson's tests executed separately for comparisons of the donor-specific profiles collected before and after reperfusion. Only genes with the expression level of at least 0.01RPKM were taken into account. Person's correlation test was also used for comparing expression landscapes before and after reperfusion across two kidneys. p value and the correlation were calculated by python scipy package.

To identify differentially regulated genes in reperfusion cases compared with nonreperfusion samples, the test of Baggerly implemented in CLC Genomic Workbench was applied to the data [15]. To determine significantly expressed genes, t-tests on weighted expression proportions were used. Transcripts with fold change > 1.5 or < -1.5 (p value <0.05) were considered for further analysis as up- or downregulated between sampling conditions, respectively.

Genes detected as differentially expressed in both of studied kidneys were further explored using heatmap analysis; gene functions were interpreted using PANTHER toolkit Version 12.0 (http://www.pantherdb.org/tools).

Pathway Studio software (Elsevier, Rockville, MD) that is able to dynamically create and draw protein interaction networks and pathways was employed for building various networks and performing Gene Set Enrichment Analysis.

## 3. Results

### 3.1. Reperfusion Does Not Lead to Substantial Change in Overall Landscape of Kidney Transcription

For each pair of samples, correlation analyses were performed by taking into consideration all genes expressed at levels of at least 0.01 RPKM in each specimen. When paired statistical tests of the differential gene expression were performed for ischemic samples taken from two different kidneys, the Pearson's correlation of expression profiles between two samples was at R= 0.89 (p value < 2.2e-16). A substantial upregulation of the genes involved in detection of chemical stimuli, olfactory sensing, and ion binding, probably reflecting intrinsic differences in the functioning of Organic Anion Transporters (OATs) [16], was detected in Kidney 2. For two reperfused specimens, similarly calculated correlation was at R = 0.91 (p value < 2.2e-16). When ischemic and reperfused specimens collected from each of the kidneys were compared to each other, same-kidney samples correlated at R = 0.98, indicating that reperfusion does not lead to substantial change in overall transcription landscape.

### 3.2. Reperfusion-Specific Transcription Program in Kidney Parenchyma

**Figure 1:**
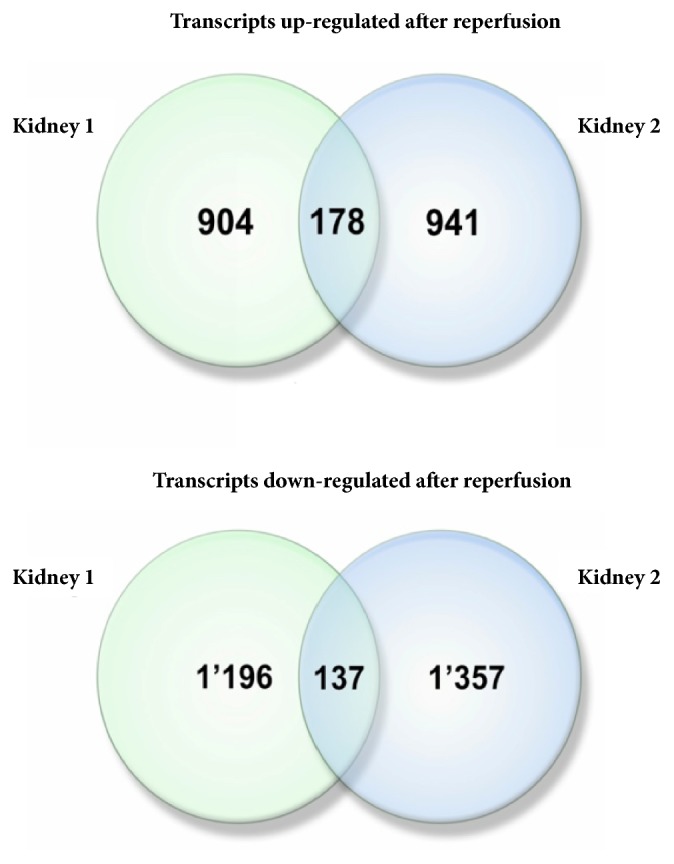
**Venn diagram of differentially expressed gene sets.** Venn diagram of gene sets differentially expressed after reperfusion in Kidney 1 and Kidney 2 and an intersection of gene sets unidirectionally overexpressed and downregulated after reperfusion in Kidney 1 and Kidney 2.

A comparison of ischemic and reperfused specimens collected from each of two kidneys yielded comparably sized sets of differentially expressed mRNAs. For Kidney 1, changes in expression levels with cut-off of 1.5 folds were detected for 2,415 genes (upregulated: N=1,082 genes; downregulated: N=1,333). For Kidney 2 at same cut-off, the list of differentially expressed transcripts included 2,613 mRNAs (upregulated: N=1,119 genes; downregulated: N=1,494). Venn diagrams reflecting the genes unidirectionally and significantly changing their expression levels in two sets of specimens are presented at Figure 1, with 178 commonly upregulated and 137 downregulated genes observed.

Common up- and downregulated genes were analyzed for relative representation of Gene Ontology (GO) terms by PANTHER. The distribution of GO functions revealed that most upregulated genes were predominantly involved in protein binding (92 genes, P< 0.0373), followed by fifteen different functional categories of genes encoding products with various types of DNA-binding activities (group p-values ranging from P < 0.00000147 to 0.0249). When all genes encoding DNA-binding proteins (N=24) were combined together, enrichment for this generalized category was detected at p< 0.000424. Remarkably, among the transcripts downregulated after the reperfusion, no enrichments for any functional category were detected, despite successful Ensemble ID-guided recognition of 75 out of 137 differentially expressed transcripts.

**Figure 2:**
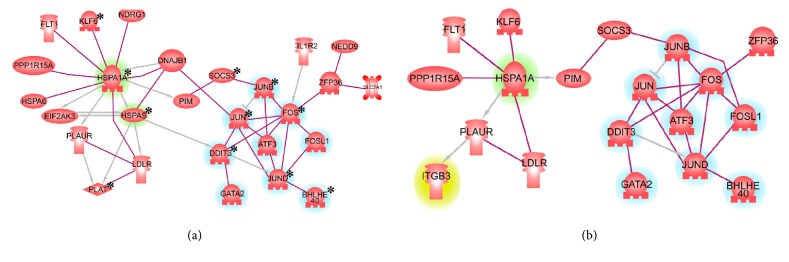
**Highly connected networks built using reperfusion-upregulated mRNAs coding for proteins known to interact with human proteins.** (a) Protein-Protein Interaction Network (PPIN) generated using the intersection of mRNAs sets unidirectionally overexpressed after reperfusion in Kidney 1 and Kidney 2. (b) PPIN generated using set of mRNAs detected to be overexpressed after comparing combined amounts of reads from both kidneys before and after reperfusion. Network was generated by Pathway Studio Network Building. Each node represents a protein. Network hubs are highlighted in blue (Jun/Fos/ATF hub) or green (HSP1A/ HSPA5 hub). In Figure 2(a), stars indicate the targets of transcription factors overexpressed in reperfusion. In Figure 2(b), protein ITGB3 is highlighted in yellow as the only entity added to the network as a result of read combining.

Reperfusion-upregulated genes encoding proteins known to bind other proteins were further explored with Pathway Studio Network Building tool set to display only direct binding connectivity. This analysis revealed that 27 out of 92 (29.3%) proteins form tightly knit network (Figure 2); three proteins (GADD45B, GADD45G, and CDKN2A) form an interacting triplet, and HES1/HES6 and S100A8/S100A9 form interacting duplets, while the rest of the upregulated genes (N = 58) remained unconnected. Remarkably, highly connected protein network (Figure 2) included two prominent hubs, which were centered on Jun/Fos/ATF transcription factors and HSP1A/ HSPA5 heat shock proteins.

The cut-off for enrichments analysis of GO Biological Processes associated with genes upregulated in reperfusion was selected at 9.99e-05. This analysis highlighted 24 protein entries broadly belonging to following categories: regulation of cell death (3 entries with p values ranging from 8.46E-07 to 4.75e-05); response to various external stimuli including bacteria and bacterial components and lipids and oxygen-containing compounds (8 entries with p values ranging from 2.41e-06 to 6.85e-05); and the regulation of metabolism in a broad sense, with a total of 13 entries, with positive regulation of nitrogen compound metabolic processes being highlighted by lowest detected p value of 7.15E-07.

As an additional control, same set of analyses were performed after joining two sets of samples. In this comparison, reads obtained for Kidney 1 and Kidney 2 were combined and compared to similarly combined gene expression values at reperfusion. This experiment affirmed the confidence in detection of genes upregulated after reperfusion, while providing a different set of downregulated transcripts (not shown). Thirty out of 43 reperfusion-upregulated mRNAs were also detected as mRNAs with unidirectionally and significantly changed expression levels in two sets of specimens analyzed separately (Table 1). Remarkably, 11 out of 30 most confidently detected genes were also present in the network formed by proteins directly binding each other (Figure 2(b)), with the most central nodes being preserved to larger degree than the dangling nodes. Only one additional protein, ITGB3, was added to the network as a result of combining the reads obtained from both kidneys.

### 3.3. Reperfusion-Specific Proinflammatory Expression Program Is a Subject of Positive Feedback Regulation

Separately, an analysis of mRNA targets for 24 DNA-binding proteins commonly upregulated after reperfusion was performed. Known validated targets of these transcription factors, including 18 protein complexes and 685 individual proteins or miRNAs, were pulled from the database hosted by Pathway Studio. Further downstream functional analysis of these targets showed substantial enrichments in the following Pathway Studio Ontologies: inflammatory cytokines (P<7.08E-19), extracellular matrix degradation proteins in general (7.66e-15), and matrix metalloproteinases in particular (2.66e-14), oncogenes (2.68e-14), adipokines (3.12e-10), lymphokines (5.18e-10), extracellular matrix polymerization proteins (6.52e-10), transcription factors of C/EBP family (1.31e-09), tumor suppressors (1.16e-08), and CSF-1/PDGF receptor family signals (1.89-e08). Importantly, target genes encoding proteins BHLHE40, DDIT3, FOS, HSPA1A, HSPA5, JUN, JUNB, JUND, KLF6, SOCS3, and PLAT were also mapped to highly connected networks built using reperfusion-upregulated mRNAs (Figure 2(a)). This observation possibly indicates positive feedback regulation of reperfusion program. Notably, a majority of these proteins (9 out of 10) were also preserved in highly connected network independently generated using the list reperfusion overexpressed genes detected after combining the read counts obtained for both kidneys.

Further functional analysis of 43 downstream targets, which, indeed, changed their expression levels in both kidneys (Table 1) revealed an enrichment in the following Pathway Studio Ontologies: soluble protein (P<1.66E-22), biofluids assayable substances (1.06e-11), a range of Jun/Fos related subnetworks (p values from 2.99e-10 to 1.14 e-4), Hairy/E(SPL)/Orange domain proteins (1.67e-8), and calprotectin (4.58e-7). A summary analysis of biological functions associated with these molecules showed an abundance of inflammation-related regulatory subnetworks, including cortisol in resolving inflammation (p < 4.08 e-4), AXL receptor inhibiting macrophages and dendritic cell function (p < 5.89 e-4), mast cell activation without degranulation through CXCR4 signaling (p < 1.11 e-3), ER stress (unfolded protein response) (p< 1.78e-3), and others.

### 3.4. Reperfusion-Specific Changes Expression of Noncoding RNAs in Kidney Parenchyma

**Figure 3:**
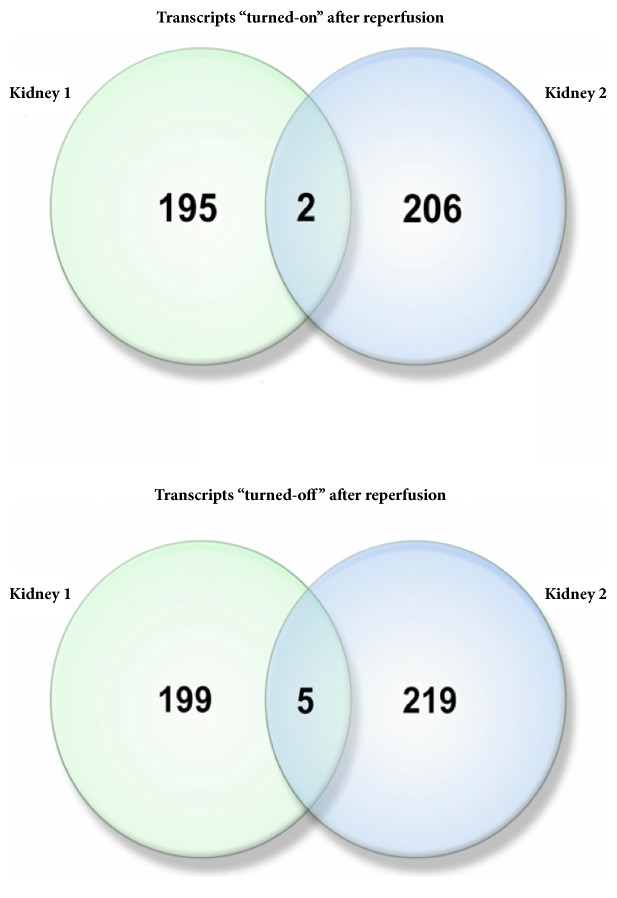
Venn diagram of gene sets awakened or silenced after reperfusion in Kidney 1 and Kidney 2 and an intersection of gene sets which changed their expression after reperfusion from nondetectable or to nondetectable.

A number of noncoding RNA transcripts unidirectionally and significantly changed their expression levels in both reperfused kidneys. Among upregulated and downregulated transcripts, there were 23.6% (42/178) and 40.1% (55/137) RNAs classified as noncoding, respectively. Additionally, in both kidneys, five noncoding transcripts were completely suppressed, while one coding and one noncoding transcript were awakened after reperfusion (Figure 3).

These transcripts were analyzed for correlations of their expression patterns in 30 human tissues with various functional groups of coding transcripts as described in [17]. For “switched-on” lncRNA RN7SL32P, top coexpression units were related to inflammatory and interferon responses and IL6/JAK/STAT3 signaling as well as allograft rejection (p < 4e-07 for each functional category). Another “switched-on” lncRNA, RP11-297M9.1, showed coexpression with various sets of genes associated with ciliary or bacterial-type flagellar motility (p values ranging from 2.5e-6 to 0.03). Importantly, lncRNA RP11-297M9.1 locates in the 3' area of protein-coding gene* GRIN2A*, whose expression increases immediately after onset of stroke [18] and being highlighted in many other publications on ischemia/reperfusion models [19–21].

Among five commonly “switched-off” RNAs, three (AC073321.3, RP11-525J21.1, and MIR1302-9) were predominantly coexpressed with genes involved in various aspects of spermatogenesis, one (RP11-659G9.3) with olfactory genes, and one (RP11-142L4.2 pseudogene) with a variety of cellular programs, including unfolded protein response and MTORC1 signaling as well as the cell cycle and its checkpoints.

## 4. Discussion

Ischemia-reperfusion injury (IRI) in transplanted organs has been a subject of many studies performed both in animal models [22–24] and in human grafts [25], with numerous biomarkers of IRI identified in blood, serum, plasma, urine, and kidney biopsies [26, 27]. From early studies of single transcripts by RT-PCR to the emergence of microarrays and, recently, RNAseq assessments of RNA profiles, the studies of expression landscapes provided a window into overall understanding of IRI in the transplanted kidneys [28–31]. In this study, we present the results of RNAseq profiling of human kidneys undergoing circulatory death-related warm ischemia (35-45 min) with subsequent extracorporeal perfusion in situ for 120 min [11].

We showed that reperfusion does not lead to substantial change in overall landscape of kidney transcription but rather activates specific program resulting in overexpression of highly connected protein network with two hubs centered on Jun/Fos/ATF transcription factors and stress response/heat shock HSP1A/HSPA5 proteins. These results align well with early observations on the role of the AP-1 dependent stress response in propagation of reperfusion injury [32, 33]. The induction of HSP proteins is a highly conserved response that protects human tissues, including renal parenchyma, from diverse physiological and environmental stressors by assisting in the refolding of denatured proteins and degradation of irreparably damaged proteins [33]. Our study indicates that the heat shock response is tightly linked to activation of the transcription factors binding to AP-1 sites. Importantly, two similarly looking HSP/AP-1 centered networks have been built independently (Figure 2(b)), indicating robustness of this finding.

We also performed an analysis of mRNA targets for 24 DNA-binding proteins commonly upregulated after reperfusion. As many validated targets of the reperfusion-upregulated transcription factors are also present in the general list of upregulated genes, we conclude that reperfusion-specific, proinflammatory expression program may be regulated by a positive feedback loop. Importantly, evidences of positive feedback regulation are seen for both nodes, namely, the heat shock response, which is generally protective against injury [33, 34], and stress-induced AP-1-dependent transcription program which may, depending on context, either contribute to injury or help in alleviation by modulating the activation of different immune cells and control cytokine expression at multiple levels [35].

In particular case of transplanted kidneys of study, the damage alleviating properties of JUN/JUNB/FOS/ATF3 network seem to prevail. The network presented at Figure 2 includes both RNA binding anti-inflammatory protein tristetraprolin, encoded by the ZFP36 gene [36] and IL-1R2, the decoy regulator of the IL-1 signaling [37]. Importantly, in context of reperfusion, the outcomes of AP-1 signaling are tied to relative expression of heat shock proteins, one of key contributors to long-term transplantation outcomes (Figure 2).

Mapping of injury-promoting and stress-protecting molecules changing their expression levels in ischemic and reperfused kidneys provides a rationale for possible organ conditioning during machine assisted ex vivo normothermic perfusion. For example, the levels of protection provided by Hsp70-like proteins could be augmented by several well-tolerated pharmacologic agents, including aspirin and geranylgeranylacetone [38], while the genes encoding proinflammatory molecules may by suppressed by siRNA-based gene-targeting approaches [39]. All types of small therapeutic molecules may be added directly to the organ preservation solution, thereby circumventing the need for injection into the bloodstream. Moreover, as the graft may be thoroughly rinsed before transplantation, the remaining cell-free siRNA and other therapeutic molecules could be removed, thus avoiding any off-target effects or systemic toxicity.

Another interesting observation made during this study was that baseline gene expression patterns in ischemic grafts may display substantial intrinsic differences. For example, baseline ischemic induction of the genes involved in detection of chemical stimuli, olfactory sensing, and ion binding was detected in Kidney 2, probably reflecting natural history of particular donor and/or intrinsic differences in the functioning of Organic Anion Transporters (OATs) [16, 40]. In Kidney 2, expression levels of OATs-encoding genes* SLC22A6* and* SLC22A8* were much higher than in Kidney 1. These OATs mediate the renal absorption and excretion of a wide range of metabolites and xenobiotics and involve elimination of uremic toxins, in particular, indoxyl sulfate, the molecular circumstance which may be relevant to subsequent functioning of the organ in the body of the recipient. Molecular subtyping of donor organs may possibly lead to the development of personalized approaches to the therapy of isolated organs within normothermic perfusion contours with individualized graft-conditioning cocktails.

## 5. Conclusion

This is the first study to profile gene expression and resultant molecular networks in kidney grafts from human donors with critically expanded warm ischemia time (WIT) before and after being reperfused in situ. Albeight very small, this study opens up a number of important lines for follow-on investigation. In particular, a study of natural diversity of the transcriptional landscapes in presumably normal, transplantation-suitable human organs is warranted. Additionally, as transplantation outcomes may be influenced by summarily outputs of the networks formed both by protective and by injury-promoting molecules, larger transcriptome-based studies of donors organs should be performed, and the resultant networks correlated with short- and long-term clinical outcomes.

## Figures and Tables

**Table 1:** **Analysis of enrichment of the lists of differentially expressed genes with known targets of transcription factors present within the same list.** Columns correspond to four independent performed runs of enrichment analysis. In each instance, relative enrichments were calculated separately for each list of differentially expressed genes: upregulated (“UP”), downregulated (“DOWN”), or merged (“UP+DOWN”).

Kidney 1		Kidney 2		Intersection of Kidney 1 and Kidney 2 datasets		Kidney 1 and Kidney 2 pooled together	
UP	DOWN	UP	DOWN	UP	DOWN	UP	DOWN

N=62	N=41	N=87	N=13	N=40	N=3	N=15	N=0
P < 9.73e-06	NS	P < 1.65e-13	P <6.39e-10	P < 3.86e-22	NS	P < 2.99e-12	NS

UP+DOWN		UP+DOWN		UP+DOWN		UP+DOWN	

N=103		N=100		N=43		N=15	
P < 1.17e-3		P <1.15e-2		P < 3.12e-15		P < 7.66e-10	

## Data Availability

The data used to support the findings of this study are available from the corresponding author upon request.

## Supplementary Materials

Supplementary 1 Supplementary Table 1. RNA sequencing data: results of the read mapping.

Supplementary 2 Supplementary Table 2. The lists of genes identified as upregulated in reperfused samples.

Supplementary 3 Supplementary Table 3. The lists of genes identified as downregulated in reperfused samples.

## References

[1] Gambato M., Frigo A. C., Castro K. I. R. (2013). Who fares worse after liver transplantation? impact of donor and recipient variables on outcome: Data from a prospective study. *Transplantation*.

[2] Kwon H., Kim Y. H., Choi J. Y. (2016). Analysis of 4000 kidney transplantations in a single center: Across immunological barriers. *Medicine (United States)*.

[3] Gondos A., Dohler B., Brenner H., Opelz G. (2013). Kidney graft survival in europe and the united states: Strikingly different long-Term outcomes. *Transplantation*.

[4] Kosieradzki M., Rowiński W. (2008). Ischemia/reperfusion injury in kidney transplantation: mechanisms and prevention. *Transplantation Proceedings*.

[5] Coulson M. T., Jablonski P., Howden B. O., Thomson N. M., Stein A. N. (2005). Beyond operational tolerance: Effect of ischemic injury on development of chronic damage in renal grafts. *Transplantation*.

[6] Heylen L., Jochmans I., Samuel U. (2018). The duration of asystolic ischemia determines the risk of graft failure after circulatory-dead donor kidney transplantation: A Eurotransplant cohort study. *American Journal of Transplantation*.

[7] Ali S., Sheerin N. S. (2013). Biomarkers of acute injury: Predicting the long-term outcome after transplantation. *Kidney International*.

[8] Chen G., Wang C., Ko D. S.-C. (2017). Comparison of outcomes of kidney transplantation from donation after brain death, donation after circulatory death, and donation after brain death followed by circulatory death donors. *Clinical Transplantation*.

[9] Cantafio A. W., Dick A. A. S., Halldorson J. B., Bakthavatsalam R., Reyes J. D., Perkins J. D. (2011). Risk stratification of kidneys from donation after cardiac death donors and the utility of machine perfusion. *Clinical Transplantation*.

[10] Hameed A. M., Pleass H. C., Wong G., Hawthorne W. J. (2016). Maximizing kidneys for transplantation using machine perfusion: From the past to the future: A comprehensive systematic review and meta-analysis. *Medicine (United States)*.

[11] Reznik O. N., Skvortsov A. E., Reznik A. O. (2013). Uncontrolled Donors with Controlled Reperfusion after Sixty Minutes of Asystole: A Novel Reliable Resource for Kidney Transplantation. *PLoS ONE*.

[12] Pesce F., Pathan S., Schena F. P. (2013). From-omics to personalized medicine in nephrology: Integration is the key. *Nephrology Dialysis Transplantation *.

[13] Salvadori M., Tsalouchos A. (2017). Biomarkers in renal transplantation: An updated review. *World Journal of Transplantation*.

[14] Rovcanin B., Medic B., Kocic G., Cebovic T., Ristic M., Prostran M. (2016). Molecular dissection of renal ischemia-reperfusion: Oxidative stress and cellular events. *Current Medicinal Chemistry*.

[15] Baggerly K. A., Deng L., Morris J. S., Aldaz C. M. (2003). Differential expression in SAGE: accounting for normal between-library variation. *Bioinformatics*.

[16] Nigam S. K., Bush K. T., Martovetsky G. (2015). The organic anion transporter (OAT) family: A systems biology perspective. *Physiological Reviews*.

[17] Perron U., Provero P., Molineris I. (2017). In silico prediction of lncRNA function using tissue specific and evolutionary conserved expression. *BMC Bioinformatics*.

[18] Saenger A. K., Christenson R. H. (2010). Stroke biomarkers: Progress and challenges for diagnosis, prognosis, differentiation, and treatment. *Clinical Chemistry*.

[19] Navarro-Sabaté À., Peralta C., Calvo M. N. (2006). Mediators of rat ischemic hepatic preconditioning after cold preservation identified by microarray analysis. *Liver Transplantation*.

[20] Andreeva K., Zhang M., Fan W. (2014). Time-dependent gene profiling indicates the presence of different phases for ischemia/reperfusion injury in retina. *Ophthalmology and Eye Diseases*.

[21] Wang Y., Liu H., Lin Y. (2017). Network-Based Approach to Identify Potential Targets and Drugs that Promote Neuroprotection and Neurorepair in Acute Ischemic Stroke. *Scientific Reports*.

[22] Speir R. W., Stallings J. D., Andrews J. M., Gelnett M. S., Brand T. C., Salgar S. K. (2015). Effects of Valproic acid and dexamethasone administration on early bio-markers and gene expression profile in acute kidney ischemia-reperfusion injury in the rat. *PLoS ONE*.

[23] Liu J., Krautzberger A. M., Sui S. H. (2014). Cell-specific translational profiling in acute kidney injury. *The Journal of Clinical Investigation*.

[24] Malagrino P. A., Venturini G., Yogi P. S. (2017). Proteome analysis of acute kidney injury – Discovery of new predominantly renal candidates for biomarker of kidney disease. *Journal of Proteomics*.

[25] Oda T., Ishimura T., Yokoyama N., Ogawa S., Miyake H., Fujisaw M. (2017). Hypoxia-Inducible Factor-1*α* Expression in Kidney Transplant Biopsy Specimens After Reperfusion Is Associated With Early Recovery of Graft Function After Cadaveric Kidney Transplantation. *Transplantation Proceedings*.

[26] Liu J., Kumar S., Dolzhenko E. (2017). Molecular characterization of the transition from acute to chronic kidney injury following ischemia/reperfusion. *JCI Insight*.

[27] Cavaillé-Coll M., Bala S., Velidedeoglu E. (2013). Summary of FDA workshop on ischemia reperfusion injury in kidney transplantation. *American Journal of Transplantation*.

[28] Mengel M., Chang J., Kayser D. (2011). The molecular phenotype of 6-week protocol biopsies from human renal allografts: Reflections of prior injury but not future course. *American Journal of Transplantation*.

[29] Mueller T. F., Reeve J., Jhangri G. S. (2008). The transcriptome of the implant biopsy identifies donor kidneys at increased risk of delayed graft function. *American Journal of Transplantation*.

[30] Halloran P. F., Famulski K., Reeve J. (2015). The molecular phenotypes of rejection in kidney transplant biopsies. *Current Opinion in Organ Transplantation*.

[31] Kurian S. M., Velazquez E., Thompson R. (2017). Orthogonal Comparison of Molecular Signatures of Kidney Transplants With Subclinical and Clinical Acute Rejection: Equivalent Performance Is Agnostic to Both Technology and Platform. *American Journal of Transplantation*.

[32] Bardella L., Comolli R. (1994). Differential expression of c-jun, c-fos and hsp70 mRNAs after folic acid and ischemia-reperfusion injury: Effect of antioxidant treatment. *Experimental Nephrology*.

[33] Nayak Rao S. (2016). The role of heat shock proteins in kidney disease. *Journal of Translational Internal Medicine*.

[34] O'Neill S., Hughes J. (2014). Heat-shock protein-70 and regulatory T cell-mediated protection from ischemic injury. *Kidney International*.

[35] Hess J., Angel P., Schorpp-Kistner M. (2004). AP-1 subunits: quarrel and harmony among siblings. *Journal of Cell Science*.

[36] Andrianne M., Assabban A., La C. (2017). Tristetraprolin expression by keratinocytes controls local and systemic inflammation. *JCI Insight*.

[37] Newton R. (2014). Anti-inflammatory glucocorticoids: Changing concepts. *European Journal of Pharmacology*.

[38] Sõti C., Nagy E., Giricz Z., Vígh L., Csermely P., Ferdinandy P. (2005). Heat shock proteins as emerging therapeutic targets. *British Journal of Pharmacology*.

[39] Glebova K., Reznik O. N., Reznik A. O. (2014). siRNA technology in kidney transplantation: Current status and future potential. *BioDrugs*.

[40] Matsuzaki T., Watanabe H., Yoshitome K. (2007). Downregulation of organic anion transporters in rat kidney under ischemia/reperfusion-induced qacute renal failure. *Kidney International*.

